# Evaluation in alcohol use disorders – insights from the nalmefene experience

**DOI:** 10.1186/s12916-016-0664-9

**Published:** 2016-08-18

**Authors:** Florian Naudet, Clément Palpacuer, Rémy Boussageon, Bruno Laviolle

**Affiliations:** 1 Meta-Research Innovation Center at Stanford (METRICS), Stanford University, 1070 Arastradero Road, Palo Alto, CA 94304 USA; 2 INSERM Centre d’Investigation Clinique 1414, Centre Hospitalier Universitaire de Rennes, Rennes, France; 3 Département de Médecine Générale, Faculté de Médecine de Poitiers, Poitiers, France

## Abstract

Nalmefene was the first treatment approved by the European Medicines Agency for reducing alcohol consumption in adult patients with alcohol dependence. It is often presented as a paradigm shift in therapeutics, but major issues limit the interpretation of the evidence supporting its use. The randomised trials submitted provided no evidence of harm reduction, the differences on consumption outcomes were of questionable clinical relevance, the target population was defined a posteriori and the drug was compared to a placebo although naltrexone was already used off-label. No post-approval randomised study is currently designed to clearly address these issues. In addition, nalmefene trials have been uncritically cited, even in guidelines. This experience reveals weaknesses in drug evaluations in alcohol dependence, which call for changes. We propose to dispense with alcohol consumption as a surrogate outcome, to consider comparative effectiveness issues, and to recommend randomised post-approval studies in case of controversial approval.

**Electronic supplementary material:**

The online version of this article (doi:10.1186/s12916-016-0664-9) contains supplementary material, which is available to authorized users.

On 13 December 2012, nalmefene, an opioid antagonist, was approved by the European Medicines Agency (EMA) for the reduction of alcohol consumption in adult patients with alcohol dependence, a high drinking-risk level, no physical withdrawal symptoms and not requiring immediate detoxification. It is the first treatment in this indication when the usual aim in alcohol dependence is abstinence from drinking. The approval [1] was based on the results of two phase III randomised controlled trials (RCTs) lasting 6 months [2, 3], one lasting 1 year [4], and four earlier RCTs including dose–response studies (two of these were unpublished studies) [5, 6]. Some additional supporting evidence was also presented, including pooled subgroup analyses [7] and further analyses on the expected harm reduction (alcohol-related physical health outcomes, injuries or social consequences) based on the literature data and on modelling from the clinical trial data [8, 9]. Despite this body of evidence, the approval was contested, with some disagreement between and within the different health authorities. For instance, six members of the Committee for Medicinal Products for Human Use at the EMA expressed divergent positions in an appendix to the assessment report. The National Institute for Care and Excellence (NICE) in the UK initially recommended nalmefene as a possible treatment for alcohol dependence [10] but subsequently distanced itself from this earlier advice [11]. The German and Swedish health authorities simply stated that there was no added benefit from nalmefene [12, 13].

The RCTs performed have thus failed to demonstrate an unequivocal benefit for the drug, despite the fact that, from a regulatory perspective, this should be their principal aim.

We propose here to examine the evidence that led to the approval of nalmefene and to understand why studies were not unequivocal in this specific case, how their results were integrated into the health authority decisions and how the controversy spread in the medical literature. Our final purpose is to propose relevant changes concerning therapeutic evaluation in the field of alcohol dependence.

## Search strategy and selection criteria

**Fig. 1:**
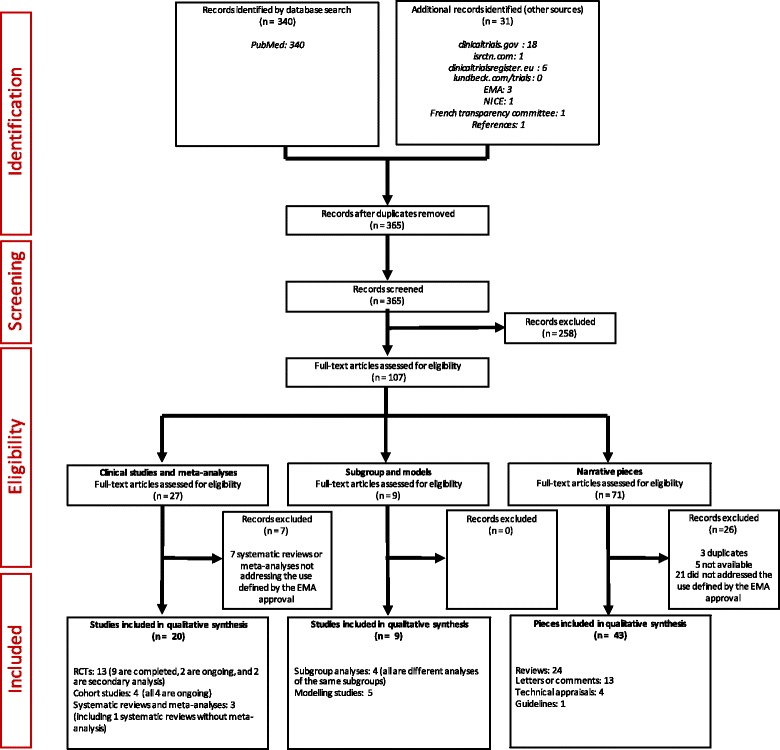
Preferred Reporting Items for Systematic Reviews and Meta-Analyses (PRISMA) diagram detailing the study selection process. *EMA* European Medicines Agency *NICE* National Institute for Health and Care Excellence

In this review of published and unpublished literature, we looked for evidence, including clinical studies (completed, terminated or ongoing), supportive studies (subgroup analyses and models) and commentaries discussing the material used for the EMA approval (letters and systematic or non-systematic reviews) about the efficacy, effectiveness or efficiency of nalmefene, via a search of PubMed, clinicaltrials.gov, isrctn.com, clinicaltrialsregister.eu and lundbeck.com/trials, up to and including 25 April 2016, using the term ‘nalmefene’. In addition, we used approval documents from the EMA, the NICE, the French transparency committee, and a earlier systematic review and meta-analysis by our team [14]. References were also identified by searching the bibliographies of relevant publications. A flow diagram detailing this process is presented in Fig. 1. All references are provided in Additional file 1.

## Evidence of a small reduction in alcohol consumption without any evidence of ‘harm reduction’

When the whole body of evidence derived from RCTs is considered, no evidence is found in the meta-analyses currently available to support the use of nalmefene for harm reduction among people treated for alcohol dependency [14]. Compared to a placebo, there were no significant differences either on mortality or on quality of life. Additionally, some crucial endpoints, such as accidents, injuries and somatic alcoholism complications, were not directly measured as specific outcomes in the trials, but were included in the composite outcome of ‘serious adverse events’, which were found to be similar across groups (although there were more adverse events in the nalmefene group). In its report, the EMA noted that, from the available data, nalmefene does not appear to raise the incidence of accidents and falls in the target population of alcohol-dependent patients [1]. However, an approved treatment should aim to reduce accidents and falls, rather than merely avoid increasing them.

When alcohol consumption outcomes were considered, there were significant reductions in heavy drinking days and total alcohol consumption in the populations studied. The evidence of the usefulness of nalmefene is thus based on the assumption that reducing alcohol consumption in alcohol-dependent patients will impact health outcomes. While there is no high-quality randomized evidence concerning the efficacy of managed alcohol programmes on their own on these health outcomes [15], some authors claim that this surrogate outcome is valid, because it is assumed that reducing alcohol consumption is useful in alcohol dependence. For example, numerous epidemiological studies have suggested [16, 17] that high consumption of alcohol is associated with an increased risk for liver disease in comparison with lower consumption. Therefore, it is tempting to assume that if individuals reduce their consumption, they reduce their levels of risk accordingly. But even if we consider that epidemiological evidence is sufficiently plausible to legitimize a decrease in consumption as a valid goal, it is not known what real reductions, in term of quantity as well as in terms of duration, might be associated with harm reduction. In addition, the benefit of a given reduction could be very different depending on the initial consumption level of a given individual (the higher his or her consumption, greater the benefit is expected to be) [18].

Furthermore, in the case of nalmefene, the reductions observed in the consumption outcomes compared to placebo were of questionable clinical significance (i.e. effect size of 0.2 for total alcohol consumption) [14], as noted by the EMA report [1]. In addition, the fuzziness surrounding the definition of these consumption outcomes highlights another problem concerning nalmefene trials. A recent paper suggested that the precise definition of the reductions in consumption (heavy drinking days and total alcohol consumption) were added after data collection ended [19].

## An attrition bias cannot not be ruled out

Attrition is common in alcohol clinical trials and missing data are an important methodological problem [20]. The nalmefene trials were no exception. All three pivotal studies had missing data for more than 35 % of the patients. Missing data prevent good-quality intention-to-treat analyses, and can cause biased estimates of the treatment effect. Additionally, there were more withdrawals, including more withdrawals for safety reasons, in the nalmefene group than in the placebo group in the 6-month and 1-year studies [14]. In addition to a possible unblinding of treatment allocation, this exposes these studies to an attrition bias. Additionally, the EMA noted that the differences in treatment effect between nalmefene and placebo, and the reduction in alcohol consumption in terms of reduction in heavy drinking days and total alcohol consumption, were inconsistent across the various sensitivity analyses [1].

## No RCT performed in the target population defined by the EMA

**Fig. 2:**
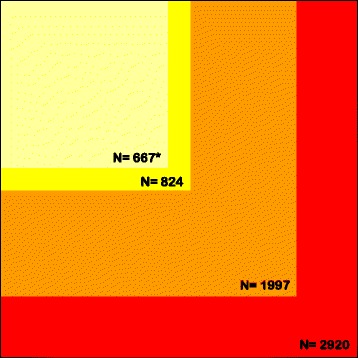
Number of patients enrolled in subgroup analyses in comparison with all patients randomised to nalmefene or placebo identified in a previous systematic review and meta-analysis [14]. *Red square* represents all randomised patients. *Orange square* represents all patients included in the three pivotal studies. *Yellow square* represents the population indicated for the use of nalmefene in the three pivotal studies. *Light yellow square* represents the population indicated for the use of nalmefene in the two 6-month pivotal studies. The subgroup analysis used for nalmefene approval was based on this population. * A publication [7] reports 667 patients whereas another publication [9] reports 641 patients for the two 6-month pivotal studies

As stated in the EMA assessment report, because there was a degree of uncertainty regarding the precise magnitude of the beneficial effects (or which analytical method was best suited to measuring it) and its clinical relevance in the total population, and in order to substantiate the clinical efficacy and the clinical relevance of nalmefene by defining a population where the benefit of nalmefene would be greatest, subgroups analyses were performed a posteriori [1]. These secondary analyses of the pivotal studies included patients with a high or very high drinking risk level (DRL) at baseline and who maintained a high or very high DRL at randomisation in two [7] or three [9] of the pivotal studies. Analyses from only two studies were presented in the EMA report. This amounts to <25 % of the existing randomised evidence, as shown in Fig. 2, which presents the numbers of patients enrolled in these subgroup analyses in comparison with all randomised patients in RCTs identified in a previous meta-analysis [14]. Further to this, subgroup analyses are generally considered explanatory rather than confirmatory, especially when performed a posteriori [21, 22]. Because it was a crucial point in the approval, the EMA mandated a scientific advisory group to deal with this issue, and concluded that while post hoc analyses were not ideal, they were commonly used in clinical trials for psychiatric drugs, given the high dropout rates encountered in these populations [1]. The fact nevertheless remains that there is no RCT performed in the specific population defined by the EMA approval. In addition, it is not obvious that doctors will be able to accurately select this target population in a real-life setting [23]. Thus, the scientific advisory group specifically advised that, to avoid misleading clinicians and to minimise off-label use, the therapeutic indications should clearly inform physicians (including general practitioners) to enable them to readily recognise the patients who could be a target for the drug [1]. This issue is central. The market of people who are continuing to drink is potentially vast, especially as the drug is likely to be used outside of the subgroup it has been recommended for owing to difficulty in recognising this group and pressure from patients and healthcare staff. For example, in France, to ensure that the use defined by the approval will be complied with as best as possible, the French transparency committee required the prescription of the drug to be restricted to specialists. Interestingly, the French minister of health decided not to follow this advice and made possible the prescription of the drug by general practitioners, arguing that alcohol dependence is an important public health issue [24].

## Evidence for the effectiveness of the drug is based on statistical models

When the subgroups were considered, even if differences were found in terms of consumption outcomes [7] and in terms of quality of life [25], there was still no evidence of harm reduction. But a reduction in consumption evidenced in 6-month and 1-year studies seems highly unlikely to be clinically relevant. The harm reduction approach is based on models that are basically an extrapolation of the results observed in subgroup analyses beyond the trial time horizon [23]. This was therefore a very rash hypothesis. The authors of these analyses conclude that the differences between nalmefene and placebo on consumption outcomes could reflect considerable effectiveness in terms of health outcomes. There is no better example of this than their estimated reduction in all-cause mortality risk of 8 % (95 % confidence interval 2–13 %) at 9 years [9]. This positive outcome was calculated using data from the subgroup analyses of the pivotal studies combined with the risks for all-cause mortality observed in meta-analyses in observational studies that included people with alcohol dependence. Other decision models using Markov chains compared costs and effects of nalmefene over 5 years. These models, also based on subgroups, supported the efficacy of nalmefene with substantial public health benefits [8], including a reduction in productivity losses and crime events attributable to alcohol [26]. They also suggested that it was a highly cost-effective treatment option [27]. These models were central to the EMA approval, because the scientific advisory group focused on the model showing that even a moderate decrease in drinking levels might be associated with a decrease in both harmful events (e.g. mortality rates, accidents) and in the relative risk of the medical outcomes typically linked to excessive alcohol drinking (e.g. liver cirrhosis), before confirming that, however modest, the effect size of nalmefene was clinically meaningful [1]. Nevertheless, these cost-effectiveness analyses were described by another group as subject to considerable uncertainty, particularly because they failed to address comparative effectiveness issues, including comparison with appropriate psychosocial support and/or a relevant active pharmacological comparator, such as naltrexone [11].

## No RCT versus an active comparator

Nalmefene is a 6-methyl derivative of naltrexone [28]. The two compounds are both opioid antagonists and are thus very similar [29]. But while naltrexone has long had an approval in the indication ‘maintaining abstinence after alcohol detoxification’, nalmefene was developed for approval in the indication of ‘reducing alcohol consumption’. Rather than evaluating an innovative compound, the nalmefene phase III programme evaluated a rather older option in a relatively new indication, while comparison with an active comparator was not warranted, because there is no official comparator. This nonetheless goes against clinical practice and beyond any pharmacological rationale. Naltrexone has been widely used off-label in this indication and there has been evidence that it is of interest for patients aiming to reduce heavy drinking days [30]. Unsurprisingly, the estimated effect size for naltrexone in this indication is small (~0.15–0.2), as is the estimated effect size observed with nalmefene [14, 31]. Nevertheless, from an ethical perspective, there is the difficult question of whether a treatment that has some previous evidence for this use without having obtained approval should be the comparator of choice.

Currently, the best available evidence for a difference between the two compounds comes from indirect meta-analyses. While the German Health authority criticised analyses of this nature [13], a recent study compared nalmefene and naltrexone indirectly and concluded that nalmefene was superior, at least on outcomes related to quantity of drinking [32]. However, in this study, subgroup analyses on nalmefene RCTs were compared with naltrexone RCTs as a whole, resulting in a violation of the similarity assumption that is necessary in indirect meta-analyses [33]. Table 1 presents the results of this analysis and results of an appropriate re-analysis where no difference was found between the two drugs.

**Table 1:** Direct (nalmefene versus placebo and naltrexone versus placebo) and indirect (nalmefene versus naltrexone) meta-analyses concerning change from baseline in quantity of drinking

Studies	Abstinence criterion	Consumption criterion	Soyka et al. analysis	Analysis of complete data
			Quantity of drinking	Quantity of drinking
			SMD [95 % CI]	SMD [95 % CI]
CPH-101-0801^a^	No more than 14 days	At least 18 heavy drinking days in the last 12 weeks	−0.48 [−0.87; −0.09]	−0.21 [−0.46; 0.03]
ESENSE 1^a^ [3]	No more than 14 days	At least 40 g alcohol/day for men and 20 g alcohol/day for women and ≥6 heavy drinking days in the last 4 weeks	−0.46 [−0.74; −0.17]	−0.35 [−0.56; −0.14]
ESENSE 2^a^ [2]	No more than 14 days	At least 40 g alcohol/day for men and 20 g alcohol/day for women and ≥6 heavy drinking days in the last 4 weeks	−0.25 [−0.52; 0.02]	−0.15 [−0.34; 0.04]
SENSE^a^ [4]	No more than 14 days	>6 heavy drinking days in the last 4 weeks	−0.36 [−0.76; 0.03]	−0.12 [−0.34; 0.1]
Nalmefene versus placebo, direct comparison, fixed-effect model			−0.37 [−0.53; −0.21]	−0.21 [−0.31; −0.10]
Anton (1999) [45]	At least 5 days	5 or more drinks per day in the last 30 days	−0.35 [−0.69; −0.01]	−0.35 [−0.69; −0.01]
Balldin (2003) [46]	No more than 14 days	At least 20 heavy drinking days in the last 60 days	0.01 [−0.35; 0.37]	0.01 [−0.35; 0.37]
Anton (2005) [47]	At least 5 days	Average consumption of at least 5 standard drinks per day for men and 4 for women in the past 90 days	−0.18 [−0.49; 0.13]	−0.18 [−0.49; 0.13]
Kranzler (2000) [48]	At least 3 days and no longer than 28 days	Not specific	0.02 [−0.33; 0.37]	0.02 [−0.33; 0.37]
O’Malley (2008) [49]	At least 4 days and no more than 30 days	More than 14 drinks (women) or 21 drinks (men) per week and at least 2 heavy drinking days during a 30-day period within the 90 days prior to baseline.	0.08 [−0.39; 0.55]	0.08 [−0.39; 0.55]
Naltrexone versus placebo, direct comparison, fixed-effect model			−0.11 [−0.27; 0.05]	−0.11 [−0.27; 0.05]
Nalmefene versus Naltrexone, indirect comparison, fixed-effect model			−0.26 [−0.04; −0.49]	−0.10 [−0.29; 0.10]
Heterogeneity in the network			I^2^ = 0 %. Q = 5.9. *p* = 0.65	I^2^ = 0 %. Q = 6.4. *p* = 0.50

^a^For those studies. Soyka et al. report results obtained in the subgroup analyses (patients concerned by the market approval) while this precaution was not taken in the naltrexone trials. Results based upon this dataset are presented in the ‘Soyka et al. analysis’ column. Results based upon the complete nalmefene studies are reported in the ‘Analysis of complete data’ columns

All analyses were performed using the frequentist approach, which is implemented in R in the netmeta library. Details about two important inclusion criteria of the included studies (abstinence and previous consumption) are given to be informative about the similarity assumption that is necessary to interpret results of indirect comparisons

## Post-approval evidence cannot address the critical issues

One might expect that, even if the approval was controversial, post-marketing data are necessary to confirm the interest or otherwise of nalmefene. Among the six currently ongoing registered studies we have identified, (1) two are RCTs versus placebo (NCT02752503 and NCT02364947) but neither is performed in the target population defined by the EMA; (2) two are non-randomised studies (NCT02382276 is a continuation study of NCT02364947, NCT02197598 being conducted among cirrhotic patients), and (3) two are non-randomised studies in ‘real-life’ European clinical settings (NCT02492581, NCT02195817). Both are prospective cohorts: the first was designed on request from the French Transparency Commission and the second was implemented in primary care but was terminated early as a result of enrolment problems. Because of their non-randomised design, these two studies will neither confirm the efficacy of the drug in its target population, nor provide evidence of comparative effectiveness.

## Dissemination of these issues in the literature

**Fig. 3:**
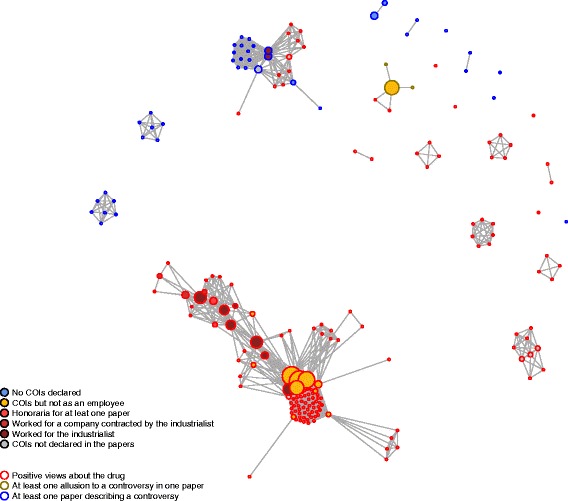
Nalmefene co-authorship researcher networks. Each *circle* represents one author (larger diameter indicates a larger number of publication by this author) and each *line* connecting two authors indicates the presence of at least one publication they have co-authored (larger diameter indicates a larger number of publication in common). As indicated in the figure, the *colours of the circles* indicate conflict of interest (*COIs*) declared in the considered papers, and the *colour of the outline* indicates the authors’ views about the drug. All analyses were performed using the igraph library in R. All identified references were included excepted four references with no authors listed

Even if these major limitations to the interpretation of the trials have been raised by the different evaluations, the nalmefene trials have been uncritically cited in the subsequent literature. Among narrative reviews, 75 % failed to present the topic as controversial (17 % allude to it without any details and only 8 % present it as controversial), and 54 % referred to the novelty of the drug describing a ‘new’ [34] ‘approach’ [35] or ‘drug’ [36] or ‘target’ [37]. Some authors also refer to ‘a paradigm shift’ and a ‘historical step in the advancement of alcohol use disorder treatment’ [38]. The 2015 Recommendations of the French Alcohol Society, issued in partnership with the European Federation of Addiction Societies, recommends nalmefene as the first-line medication for reducing alcohol consumption in subjects with alcohol dependence, without mentioning the subgroup of patients defined by the EMA approval [39]. Links between authors, addiction societies and pharmaceutical companies have been pointed to as an explanation for these uncritical opinions [40]. While it seems impossible to determine whether conflicting interests or strong allegiances have distorted the interpretation of the evidence, a co-authorship diagram illustrates the various links disclosed in the papers we have considered (Fig. 3). It shows (1) a first cluster, with authors closely related to the industry (as most were involved in the development programme) who expressed positive views about the drug; (2) a second cluster involving authors of earlier studies, some of whom expressed some concern about the usefulness of the drug (in the context of interpretation of a negative study); and (3) a constellation of small clusters or isolated authors including the few contributions presenting the controversial issues (mostly letters to editors), the authors of which rarely disclosed conflicting interests.

## Discussion

The key issue is whether nalmefene use is based on anything more than a clever use of the data to get an existing drug licensed for a new indication, or whether it is really a new way of seeing things. Various authors seem enthusiastic about the concept of doing away with the need to aim for abstinence from drinking, and proposing cutting down consumption as being a valid goal. But even if this is genuinely a new paradigm in alcohol treatment, nalmefene was evaluated using the traditional paradigm, and appears to fall short of the evidence required. Indeed, the use of surrogate outcomes, the questionable clinical relevance of the differences evidenced, the use of a posteriori subgroup analyses, and the inappropriate comparison with placebo when another treatment was available are all reasons that could explain why the results of nalmefene RCTs cannot be interpreted as confirmatory. All of this leads to persistent uncertainty, and to difficult and controversial decisions for the health authorities, and is liable to generate another public health problem, because care that is offered to patients is liable to be questioned and discredited.

In addition, and from a clinical perspective, marketing a drug for people who continue to drink (especially if it is ineffective) may have seriously detrimental psychological and social implications. The licensing of nalmefene may suggest to some people that they can continue drinking when they might otherwise have come to the realisation that they need to stop. Given that people with alcohol problems are often in denial about the harmful effects of drinking, and accustomed to looking for a chemical solution for their problems, this is a real danger that may actually perpetuate harmful drinking in the long term.

Therefore, scientists and clinicians need to engage in a collaborative effort to reach an evidence-based consensus that extends beyond the case of nalmefene and concerns all regulation of medication for alcohol dependence. In our opinion, this implies changes in our current research paradigm. First, we propose doing away with alcohol consumption as a surrogate for treatment success. We propose instead the use of mortality and health outcomes (motor vehicle crashes, injuries and harm). Alternatively, quality of life could be considered, providing unambiguous tools are developed to asses it in an optimal way [41]. Although simple in appearance, these changes would be revolutionary in the field of alcohol use disorders, where very few trials have reported these health outcomes (or when they have done so, they had not been designed or powered to assess them correctly). A meta-analysis including 22,803 participants concluded that the evidence from trials was insufficient to draw any conclusions about improved health outcomes attributable to pharmacotherapy [42].

One can argue that adequately designed trials of health outcomes would be unfeasible (too large and too long) and that no company would agree to finance such trials. Is it because such studies are genuinely not feasible? One should bear in mind that ‘mega-trials’ have been successful in the field of cardiovascular medicine. Or is it because the current treatments will not show any clinically relevant utility? Companies confident in their products should have no reason to avoid the test of mega-trial evidence [43]. Large, randomised, controlled cluster trials may be of interest here, including studies comparing different strategies such as abstinence and harm reduction.

Second, we propose that the evaluation of therapies should be collaborative and based on coherent agendas where all compounds of interest are integrated into a common research programme exploring issues of comparative effectiveness. In the current context of drug research, where (1) trials are focused on single patented compounds and designed to meet the requirements of the regulatory bodies and (2) there is a strong competition between pharmaceutical companies, a change of this sort appears utopic. Thus we propose that health authorities should be involved a priori in designing the studies that can lead to market approval in order to ensure that comparative effectiveness issues are adequately addressed, which is not the case currently, at least for the EMA [44].

Finally, we suggest that controversial approvals issued on the grounds of subgroup analyses should be compulsorily confirmed in subsequent post-approval randomised comparative studies. In case of negative results in such studies, the drug approval should be withdrawn. In our opinion, all these changes are necessary to avoid wastage of resources, investments and scientific effort, and to put an end to the persistent uncertainty that the case of nalmefene exemplifies.

Obvious perspectives are (1) to explore the prevalence of controversial approvals of the nalmefene type among all EMA approvals and (2) with the help of economists, to find alternative models where the requirement of strong evidence will not lead to risk avoidance tactics by those who evaluate the treatments.

## Abbreviations

DRL, drinking risk level; EMA, European Medicines Agency; NICE, National Institute for Health and Care Excellence; RCT, randomised controlled trial

## Additional file

Additional file 1: References. (XLSX 58 kb)
